# Efficacy of a regional systems intervention for suicide prevention (SUPREMOCOL) in Noord-Brabant, the Netherlands

**DOI:** 10.1192/j.eurpsy.2023.1179

**Published:** 2023-07-19

**Authors:** C. M. Van Der Feltz-Cornelis, I. Elfeddali, M. Metz, S. de Jong, M. Bakker, C. van Nieuwenhuizen

**Affiliations:** 1 Health Sciences, University of York, York, United Kingdom; 2 CLGG; 3 GGZ Breburg; 4 MTO; 5 Tranzo, Tilburg University, Tilburg, Netherlands

## Abstract

**Introduction:**

Worldwide, annually more than 800,000 suicides occur. In the Netherlands, suicide rates rose from 8.6 per 100,000 in 2007 to 11.4 per 100,000 in 2016. Rates in the province of Noord-Brabant were consistently higher than the national average. Noord-Brabant is a province in the south of the Netherlands covering an area of over 4700 km2 with 2.5 million inhabitants. Although Noord-Brabant has five specialised mental healthcare institutions (SMHIs), and 90% of suicides are deemed related to mental disorders, 60% of those who died by suicide did not receive mental health treatment. However, with good access to treatment, suicide could be preventable.

**Objectives:**

To evaluate whether the systems intervention compared to the regular care approach led to a reduction in suicides in Noord-Brabant. We aimed to attain a reduction in suicides of at least 20%.

**Methods:**

Co-design and development of a digital monitoring system and decision aid. Stepwise implementation per subregion of the systems intervention by the five specialized mental healthcare institutions (SMHIs) and their chain partners. Pre-post analysis for the whole province (Exact Rate Ratio Test, Poisson count).

**Results:**

The SUPREMOCOL systems intervention consisted of four pillars, which were all supported by a digital decision aid and monitoring system. This was provided via a desktop computer with a secured login. The data were kept on a secured encrypted server. The number of professionals accessing the system was limited to two per SMHI or other chain partner. They could only see patients in their subregion, not in the whole of the province. The pillars of our systems intervention for suicide prevention are:

1. Swift identication of people at risk for suicide by triage on the spot after a non-fatal suicide attempt.

*2.* Provision of swift access to specialised mental health care for those at risk

3. Accommodating transitions in care following a collaborative care approach

4. Prevention of suicidal attempts after discharge or treatment dropout by 12 months telephone follow-up.

Suicide rates dropped 17.8% (p=.013) from baseline (2017) to implementation in 2018 and 2019. This is a significant reduction (p=0.043) compared to the non-significant drop in the rest of the Netherlands. Suicide rates dropped further by 21.5% (p = 0.002) in 2021.

Noord-Brabant also dropped in the relative rank of the number of suicides, from second place in 2015 to third in 2018.

**Conclusions:**

During the SUPREMOCOL systems intervention, over a period of 4 years, there was a sustained and significant reduction of suicides in Noord-Brabant. We attained a reduction of 21,5% in total. Noord-Brabant dropped in the relative rank of the number of suicides, from second place in 2015 to third in 2018. This is a result that warrants further research and implementation into system interventions for suicide prevention with digital support such as SUPREMOCOL.

**Disclosure of Interest:**

C. Van Der Feltz-Cornelis Grant / Research support from: The Netherlands Organisation for Health Research and Development, I. Elfeddali: None Declared, M. Metz: None Declared, S. de Jong: None Declared, M. Bakker: None Declared, C. van Nieuwenhuizen: None Declared

